# Chicken meat quality: genetic variability and relationship with growth and muscle characteristics

**DOI:** 10.1186/1471-2156-9-53

**Published:** 2008-08-18

**Authors:** Elisabeth Le Bihan-Duval, Martine Debut, Cécile M Berri, Nadine Sellier, Véronique Santé-Lhoutellier, Yves Jégo, Catherine Beaumont

**Affiliations:** 1 Institut National de la Recherche Agronomique (INRA), UR83 Recherches Avicoles, F-37380 Nouzilly, France; 2 INRA, UR370 Qualité des Produits Animaux, F-63122 Saint-Genès-Champanelle, France; 3 Hubbard, F-35220 Châteaubourg, France

## Abstract

**Background:**

The qualitative properties of the meat are of major importance for poultry breeding, since meat is now widely consumed as cuts or as processed products. The aim of this study was to evaluate the genetic parameters of several breast meat quality traits and their genetic relationships with muscle characteristics in a heavy commercial line of broilers.

**Results:**

Significant levels of heritability (averaging 0.3) were obtained for breast meat quality traits such as pH at 15 min post-slaughter, ultimate pH (pHu), color assessed by lightness L*, redness a* and yellowness b*, drip loss, thawing-cooking loss and shear-force. The rate of decrease in pH early post-mortem and the final pH of the meat were shown to be key factors of chicken meat quality. In particular, a decrease in the final pH led to paler, more exudative and tougher breast meat. The level of glycogen stored in breast muscle estimated by the Glycolytic Potential (GP) at slaughter time was shown to be highly heritable (h^2 ^0.43). There was a very strong negative genetic correlation (rg) with ultimate meat pH (rg -0.97), suggesting a common genetic control for GP and pHu. While breast muscle weight was genetically positively correlated with fiber size (rg 0.76), it was negatively correlated with the level of glycogen stored in the muscle (rg -0.58), and as a consequence it was positively correlated with the final pH of the meat (rg 0.84).

**Conclusion:**

This genetic study confirmed that selection should be useful to improve meat characteristics of meat-type chickens without impairing profitability because no genetic conflict was detected between meat quality and meat quantity. Moreover, the results suggested relevant selection criteria such as ultimate pH, which is strongly related to color, water-holding capacity and texture of the meat in this heavy chicken line.

## Background

As in other animal species, the technological quality of poultry meat is now of major importance, since poultry meat is nowadays usually consumed as cuts or as processed products rather than as whole carcasses. As already reported for pigs [1], technological quality refers to several meat properties, including water-holding capacity (i.e. drip loss during storage), intensity and homogeneity of color, firmness, shelf-life and processing yields. Meat quality is closely related to the decrease in muscle pH post-mortem. Rapid postmortem decline in pH (evidenced by low pH value measured 15 min post-slaughter in poultry, i.e. pH15) results in PSE (pale, soft, exudative) meat with a pale aspect and reduced water-holding capacity [2,3]. Variations in the extent of decrease in pH are also responsible for variations in meat quality. Low ultimate pH (measured 24 h post-slaughter in poultry) results in "acid meat", with similar defects to those of PSE meat [4], while high ultimate pH leads to DFD (dark, firm, dry) meat with dark color and poor storage quality [5]. In pigs, the PSE meat and "acid meat" defects have been shown to be controlled by major genes [1], i.e. halothane sensitivity [6] and RN [7,8] genes, respectively.

The inclusion of meat quality in pig breeding schemes dates back to the 1970–1980s [1]. Varying emphasis has been given to traits of interest according to country such as meat color (certainly the most widely used quality indicator), pH and intramuscular fat content. Genetic studies on meat quality traits in poultry are more recent. Quite significant levels of heritability (ranging from 0.35 to 0.57) were obtained for meat pH, color and water-holding capacity in two studies conducted on the same experimental broiler line slaughtered under experimental conditions [9,10]. More moderate heritability values (ranging from 0.12 to 0.22) were reported for the same meat traits measured in turkeys slaughtered under commercial conditions [11]. A study performed in quails [12] also reported moderate to high levels of heritability (0.22–0.48) of ultimate meat pH and color indicators. The present study reports the first evaluations of genetic parameters of meat quality traits and their genetic correlations with growth and muscle characteristics in a commercial broiler line.

## Results

Descriptive statistics for growth and body composition traits and for muscle and meat characteristics are summarized in Table 1. Distributions of these traits were close to normality, except for drip loss (DL) for which slight asymmetry was observed (data not shown).

**Table 1:** Descriptive statistics and heritability estimates for body weight, body composition, muscle characteristics and meat quality traits.

**Traits**	**N**	**Mean ± SD**	**Min.**	**Max.**	**h^2 ^± SE**
**Growth and body composition**					
Body weight at 6 weeks (g)	592	2141 ± 326	1234	3028	0.49 ± 0.06
Weight gain from 4 to 6 weeks (g)	596	1040 ± 229	357	1646	0.30 ± 0.05
Pectoralis *major *muscle weight (g)	578	148.6 ± 28.4	53.0	231.9	0.38 ± 0.06
Breast muscle yield (%)	580	17.8 ± 1.5	11.4	22.4	0.30 ± 0.04
Abdominal fat (%)	583	2.6 ± 0.6	0.6	4.6	0.48 ± 0.06
**Muscle characteristics**					
Fiber Cross Section Area (μm^2^)	592	1831 ± 426	630	3157	0.41 ± 0.06
Lactate (μmol/g muscle)	596	33.1 ± 10.0	6.3	56.9	0.27 ± 0.05
pH 15 min *post-mortem *(pH15)	599	6.45 ± 0.13	6.02	6.79	0.30 ± 0.05
Glycolytic Potential (μmol/g muscle)	591	108.0 ± 17.7	70.0	167.2	0.43 ± 0.05
Ultimate pH (pHu)	587	5.64 ± 0.12	5.35	6.04	0.34 ± 0.06
**Meat quality traits**					
Lightness (L*)	590	54.9 ± 3.0	42.6	63.7	0.35 ± 0.05
Redness (a*)	587	-0.8 ± 0.7	-2.8	1.3	0.25 ± 0.05
Yellowness (b*)	590	11.8 ± 1.6	8.2	16.8	0.31 ± 0.06
Drip loss (DL, %)	589	1.6 ± 1.0	0.0	6.2	0.26 ± 0.04
Thawing-cooking loss (TCL, %)	581	14.6 ± 4.8	3.1	28.6	0.35 ± 0.05
Warner Bratzler shear force (WB, N/cm^2^)	570	14.5 ± 3.0	5.9	25.5	0.34 ± 0.05

### Heritability estimates

As shown in Table 1, the heritability for growth and body composition traits was moderate to high (estimates ranging from 0.30 to 0.49) in this pure broiler line. Muscle characteristics such as fiber cross section area (CSA) and GP exhibited high levels of heritability (over 0.40). The traits related to decrease in pH post-mortem (i.e. lactate, pH15 and pHu) and to meat quality (color, water retention, texture) were significantly heritable, with heritability values ranging from 0.25 to 0.35.

### Genetic correlation estimates

This study revealed a strong genetic association between breast muscle GP and pHu, with an estimated genetic correlation of -0.97 ± 0.03. Lactate concentration and pH15 were also highly negatively correlated (rg -0.88 ± 0.05). In contrast, the rate and extent of decrease in pH appeared to be genetically independent, since pH15 and pHu exhibited a genetic correlation of -0.05 ± 0.24.

As summarized in Table 2, post-mortem muscle metabolism traits were significantly genetically related to meat quality traits. In particular, pHu exhibited significant negative genetic correlation with meat lightness and yellowness (rg -0.65 ± 0.11 and -0.54 ± 0.11, respectively), and even more marked negative correlation with meat drip loss, thawing-cooking loss and Warner Bratzler shear force (rg -0.80). As expected, opposite and somewhat less pronounced genetic correlations were found between meat quality traits and muscle GP. Muscle pH15 was mainly related to lightness and drip loss of meat (rg -0.52 ± 0.10 and -0.55 ± 0.10, respectively).

**Table 2:** Estimated genetic correlations between post-mortem muscle characteristics and meat quality traits.

Meat traits^1^	Lightness (L*)	Redness (a*)	Yellowness (b*)	Drip Loss	Thawing-cooking loss	Warner Bratzler shear force
Lactate	0.28 ± 0.16	0.36** ± 0.08	0.41** ± 0.11	0.54** ± 0.04	0.20 ± 0.10	0.36** ± 0.07
pH15	-0.52 ** ± 0.10	-0.02 ± 0.15	-0.16 ± 0.18	-0.55** ± 0.10	-0.19 ± 0.10	-0.24 ± 0.14
Glycolytic Potential	0.52** ± 0.07	0.51** ± 0.11	0.60** ± 0.10	0.78** ± 0.04	0.49** ± 0.10	0.52** ± 0.05
pHu	-0.65** ± 0.11	-0.35** ± 0.13	-0.54** ± 0.11	-0.89** ± 0.05	-0.80** ± 0.10	-0.81** ± 0.06

^1 ^Lactate = Lactate concentration 15 min post-slaughter; pH15 = pH measured 15 min post-slaughter; pHu = Ultimate pH.

** Genetic correlation is significantly different from zero (p < 0.01).

Body and breast muscle weights appeared to be significantly related to fiber size, with positive genetic correlations of 0.69 ± 0.08, 0.76 ± 0.06 and 0.48 ± 0.09 between fiber CSA and weight gain (between 4 and 6 weeks), breast muscle weight and breast muscle yield, respectively. Interestingly, breast muscle weight exhibited a significantly negative genetic relationship with muscle GP (rg -0.58 ± 0.11), and in turn a positive correlation with pHu (0.84 ± 0.07). Significantly negative genetic correlations were also found between breast muscle mass and lightness (rg -0.55 ± 0.10), drip loss (-0.65 ± 0.10), thawing-cooking loss (-0.80 ± 0.06) and Warner Bratzler shear force (-0.60 ± 0.10).

## Discussion

For the first time in a commercial broiler line, this study evaluated both the contribution of genetics to variations in meat quality traits and the genetic correlations with muscle characteristics such as fiber size and glycogen content. Quite significant levels of heritability were evidenced for meat properties such as thawing-cooking loss that can affect the processability of meat, and color and toughness that can influence the sensorial quality of meat. These genetic results emphasized the importance of the decrease in muscle pH post-mortem for breast meat quality in poultry. They indicated that, as for pigs [1], the final pH has an extensive effect on the water-holding capacity, color and texture of raw and cooked meat, while the early decrease in pH mainly influences the drip loss and lightness (L*) of raw meat, at least in this genotype. Selection for a lower final pH would lead to a higher incidence of pale and exudative meat that is tough after cooking and not very appropriate for industrial processing. On the other hand, selection for a higher final pH could improve the processing yield but could also affect storage and sensorial quality because of negative influences on microbial development and juiciness of the meat [5]. Ultimate pH, lightness and drip loss of meat were introduced into the French national breeding program for pigs in the 1980s, forming a combined quality index. It has been maintained constant across the generations of selection.

The strong negative genetic correlation between glycogen content of breast muscle (estimated through the glycolytic potential) and ultimate pH represents a major result in the present study. The genetic control of glycolytic potential and its genetic relationships with meat quality have been more widely studied in pigs than in poultry. Genetic studies in pigs have focused on either post-mortem glycolytic potential (PMGP), as for the present study, or on *in vivo *glycolytic potential (IVGP) obtained from muscle biopsy on live animals (which is not yet available for the chicken). In pigs, fairly negative genetic correlations (ranging from -0.74 to -0.99) have been reported between PMGP and pHu measured on the same muscle or on different muscles with close metabolic characteristics [13]. Corresponding correlations were slightly lower when IVGP was considered [13]. Heritability values for IVGP were around 0.25 in a population of pigs without the RN^- ^allele [13], while an average value of 0.21 was reported for pHu [1]. These genetic results together demonstrated that GP and pHu have close genetic control, and that in poultry, as in pigs, both traits can be modified by selection. In agreement with a previous genetic study in an experimental broiler line [10], the present study indicated that the rate and the extent of decrease in pH post-mortem are under the control of different genes. A similar conclusion was drawn from a selection experiment in pigs, in which a very low genetic correlation was found between IVGP and pH measured 30 min post-mortem [14]. In the chicken, the rate of decrease in pH was shown to be influenced by behavior at slaughter and hastened by struggle activity of the birds on the shackle line, especially wing flapping [15]. However, little is known to date about the influence of genetics on such behavioral traits and the implications for meat quality.

By estimating the genetic correlations, this study made it possible to correlate responses on muscle and meat quality traits with selection on growth and breast development applied in meat-type chicken. These results indicated that selection for increased breast muscle mass is expected to lead to greater fiber hypertrophy, since a strong positive genetic correlation was observed between both traits. This was in agreement with previous results obtained by comparing experimental chicken lines divergently selected for growth [16], or differing in breast yield [17]. Most studies in pigs have indicated that selection for lean growth is associated with increases in both fiber size and number [18]. The extent to which fiber number can be modified to increase breast muscle mass in the chicken has still to be investigated. Our original results also indicated that (at least in this meat-type strain) selection for increased growth and breast muscle mass can be expected to reduce glycogen storage and in turn to increase ultimate breast meat pH. Similar results have been reported at the phenotype level, when experimental and commercial chicken lines selected for increased body weight and breast yield were compared to their respective unselected control lines [19]. Inverse relationships have been reported in pigs, for which carcass leanness appeared to be moderately positively correlated with muscle GP and negatively with pHu [13,1]. This suggests that physiological and genetic factors involved in the control of GP and pHu could be at least partly different between pigs and poultry.

## Conclusion

Meat quality homogeneity has become a major concern for the poultry market. This genetic study confirmed that selection could be valuable to improve meat characteristics. The major factors contributing to meat quality were heritable, and no genetic conflict was detected between meat quality and meat quantity. Furthermore, the present results suggest that the ultimate pH of meat is a relevant selection criterion since it was strongly related to meat color, water-holding capacity and texture. More research is now needed to define the optimal breeding strategy to improve meat quality, which could be based either on classical polygenic selection or on the use of molecular markers. The first Quantitative Trait Loci (QTL) of meat quality traits were recently identified in a cross between experimental chicken lines divergently selected for growth [20]. Such research has now to be extended to commercial flocks, in order to identify effective molecular tools for selection on poultry meat quality.

## Methods

### Animals, Rearing and Slaughtering Conditions

This genetic analysis was conducted on 312 male and 293 female pedigree birds, which were the progeny of 15 sires and 64 dams. The birds originated from a male grand-parent line intensively selected for growth and breast muscle yield, and currently used by Hubbard (Chateaubourg, France) to produce parent males. As described in detail by Berri et al. [21], birds were reared in two successive batches under regular conditions in a conventional poultry house at the INRA Experimental Poultry Unit (Nouzilly, France). Birds were given *ad libitum *access to a standard diet throughout the rearing period and were individually weighed every two weeks (i.e. at 2, 4 and 6 weeks). At 6 weeks of age and after 7 hours feed withdrawal, all the birds were slaughtered at the experimental processing plant of the INRA Experimental Poultry Unit. Before sacrificing by ventral neck cutting, birds were electrically stunned (125 Hz AC, 80 mA/bird, 5 s) in a water bath, bled for 3 min, and scalded at 51°C for 3 min. After removal of the gut, whole carcasses were air chilled (airflow of 7 m^3^) and stored at 2°C until the next day.

### Carcass and meat quality traits

Breast muscle (Pectoralis *major *plus *minor*) and abdominal fat weights were measured after carcass dissection, 1 day post-slaughter. Corresponding ratios were calculated in relation to live body weight at 6 weeks. All measurements for meat characteristics were performed on the *Pectoralis major *muscle. The pH at 15 min and 24 h post mortem was measured with a portable pH-meter (Model 506, Crison Instruments, SA, Spain) equipped with a xerolyte electrode. At 15 min post mortem, pH was estimated from 2 g of muscle mixed in 18 mL of a 5 mM iodoacetate solution. This method was described as a reference method by Santé and Fernandez [22]. At 24 h post mortem, the ultimate pH of meat (pH_u_) was recorded by direct insertion of the xerolyte electrode in the muscle. This method was adopted because of the significant correlation obtained 24 h post mortem between the direct tissue measurement of pH and the reference "iodoacetate" method [22]. Breast meat color was measured at 24 h post-slaughter using a Miniscan Spectrocolorimeter with the CIE L*a*b* system, where L* represents lightness, a* redness and b* yellowness. Higher L*, a* and b* values correspond to paler, redder and more yellow meat, respectively. The water-holding capacity of breast meat was estimated through drip loss (DL) measured after 2 days of storage of the fillet hung in a plastic bag and expressed as a percentage of the initial muscle weight. After DL measurement, P. *major *muscle was vacuum-packed and stored at -25°C. For meat texture analysis, breast muscle was thawed overnight at 4°C, cooked in a water-bath at 85°C for 15 min to an internal endpoint temperature of 70°C, and cooled in crushed ice for 20 min. The thawing-cooking loss was expressed as a percentage of the fresh muscle weight. The toughness of cooked meat was evaluated by the Warner Bratzler (WB) shear test using an Instron Universal Testing Instrument.

### Muscle Parameters

The P. *major *muscle glycogen, glucose-6-phosphate, free glucose and lactate concentrations (expressed in μmol/g of muscle) were measured according to Dalrymple and Hamm [23] from 1 g of fresh tissue taken and homogenized in 10 ml of 0.55 moles perchloric acid 15 min post mortem. Glycogen content available in breast muscle at slaughter time was estimated through the post mortem glycolytic potential (GP) according to the Monin and Sellier [24] equation:

GP = 2 [(glycogen) + (glucose) + (glucose-6-phosphate)] + (lactate).

GP was expressed as micromoles of lactate equivalent per gram of fresh tissue. The CSA of P. *major *muscle fibers was determined as described by Rémignon et al. [16] on 12 μm-thick cross sections stained with red azurobin. Mean CSA was determined on approximately 300 fibers in 3 random fields for each muscle.

### Genetic Parameter Estimation

Descriptive statistics for the different traits were calculated by the UNIVARIATE procedure of SAS software [25]. Genetic parameters were computed by VCE4 software using multivariate analysis and the REstricted Maximum Likelihood (REML) method [26]. The following linear mixed model was used:

**y **= **X_1_β_1 _+ X_2_β_2 _+ Zu + ε**

in which **y **is the vector of performances observed, **β_1_**and **β_2 _**the vectors of fixed effects for batch and sex, **u **the vector of genetic animal effects, and **e **the vector of residuals. **X_1_**, **X_2_**, and **Z **are the corresponding incidence matrices. As pedigree information was limited to the sires and dams of the birds measured for meat quality, the maternal environmental effects could not be correctly estimated in this genetic study. The analyses on growth performance excluded body weight measurements at the early ages of 2 and 4 weeks (which are known to be influenced by maternal effects) to focus on body weight at 6 weeks or weight gain expressed as the difference between body weight at 4 and 6 weeks.

## Authors' contributions

EBD supervised the genetic analyses and drafted the manuscript. MD supervised the experimental design and performed the genetic analyses. CMB and VSL supervised the measurements of meat and muscle characteristics. EBD, MD, CMB, VSL, NS, and CB participated in the design of the study and data collection and helped to draft the manuscript. YJ supervised the breeding of the line and the production of the pedigree chicks used for this trial, and helped to draft the manuscript. All authors read and approved the final manuscript.
